# Comparison between Continuous and Fractionated Game Format on Internal and External Load in Small-Sided Games in Soccer

**DOI:** 10.3390/ijerph17020405

**Published:** 2020-01-08

**Authors:** Luís Branquinho, Ricardo Ferraz, Bruno Travassos, Mário C. Marques

**Affiliations:** 1 Department of Sport Sciences, University of Beira Interior, 6201-001 Covilhã, Portugal; brankinho_07@hotmail.com (L.B.); bfrt@ubi.pt (B.T.); mariomarques@mariomarques.com (M.C.M.); 2 Research Centre in Sports, Health and Human Development (CIDESD), 6201-001 Covilhã, Portugal; 3 Castelo Branco Football Association, 6000-280 Castelo Branco, Portugal; 4 Portugal Football School, Portuguese Football Federation, Oeiras, 1495-433 Lisboa, Portugal

**Keywords:** soccer, training load, external load, internal load, continuous method, fractionated method

## Abstract

This study aimed to identify the effects of continuous and fractionated game formats on internal and external load in small-sided games in soccer. Twenty male professional soccer players participated in the study performing the same exercise (5 vs. 5 players) continuously (1 × 24 min) and in a repeated/fractioned manner (2 × 12 min, 4 × 6 min, and 6 × 4 min). A comparison between playing conditions was assessed by means of standardized mean differences calculated with combined variance and respective confidence intervals of 90%. The limits for the statistics were 0.2, trivial; 0.6, small; 1.2, moderate; 2.0, large; and >2.0, very large. The results indicate that the use of the continuous method seems to present the tendency of less physical impact on the internal and external loads compared to the fractionated method. In addition, the higher number of exercise repetitions in the fractionated method was found to increase the external load compared to the continuous method. This study showed that application of small-sided games by the fractionated method tends to result in higher training loads.

## 1. Introduction

Football is characterized as an intermittent sport modality involving frequent actions of high intensity, interspersed with longer or shorter recovery periods [1]. In fact, players perform average sprints of 2–4 s every 90 s during a game, highlighting the importance of anaerobic efforts for success in the game and suggesting these efforts’ characterization as long-term intermittent modality [2]. Training methods in football have evolved over the years [3], from privileged exercises without a ball which develop physical capacities to new methods and exercises which simultaneously improve physical capacities along with technical and tactical skills in accordance with the modern demands of the game [4]. Small-sided games (SSGs) have been increasingly used by coaches because of their benefits and advantages, as when properly designed they can represent an effective strategy for multi-component training [5]. Indeed, SSGs enables the development of both physical/physiological and technical/tactical skills at the same time [6], thus presenting itself as a more effective training method compared to traditional sprint training [7]. These findings have recently been corroborated by a study summarizing the effects of SSGs across 16 studies drawn from multiple sports and population types. The authors concluded that SSGs were more effective for the development of skill and endurance than traditional conditioning training and traditional sprinting training [5].

Thus, the adequate design of SSGs that stresses anaerobic efforts is paramount for promoting appropriate training stimuli according to training goals and match demands [8]. For that, coaches should be aware of the relationship between SSGs variables and the required training stimuli and training load [9,10,11].

Training load control has been described as a reliable method for monitoring training stimulus response in football [12,13] through the use of internal and external load variables that can be conditioned by the manipulation of SSGs through the number of repetitions, duration of each repetition, and duration of rest [6,7,8,9]. In addition, the impact of such manipulations on the training load in football and consequently on the aerobic or anaerobic demand of SSGs has yet to be elucidated completely and needs to be investigated [7,8,9].

The alteration of these variables can be generally understood as continuous (i.e., without repetitions or rest intervals during the exercise) or fractionated (i.e., exercise performed repeatedly and with rest intervals between repetitions) methods. In fact, the literature has described that the performance of the exercise by either continuous or fractionated methods can cause changes in the training load [9], particularly by the changes that occur in the intensity distribution during the different periods of performance [14]. However, the differences in their application are not yet clear [9,10,15,16]. In fact, few studies have investigated the effects of applying the continuous or fractionated method on SSGs, and previous results are not conclusive and differ according to the experimental design adopted. For example, Fanchini et al. [14] investigated the internal and external load associated with both fractionated methods. Based on a comparison of 2-, 4-, and 6-min fractionated exercises, the authors found higher responses to the internal training load for 4- compared to 6−min repetitions in SSGs. In rugby, Samson et al. [17] revealed that the number and duration of the repetitions affect the internal (heart rate (HR)) and external (number of displacements at high speed) load in a positive manner. Results from another study [18] suggest that the use of SSGs through the continuous method induce lower HR responses compared to the fractionated method. However, Hill-Haas et al. [19] concluded that there was a higher internal load but with a lower external load when using continuous vs. fractionated methods. In a recent study that analyzed internal and external load variations between two fractional regimes (6 × 3 min and 3 × 6 min) during SSGs, the results show that longer variations increase the perception of effort and contribute to a large decrease in total running distances and total accelerations and decelerations [20].

These data highlight that the differences in the use of both methods remain inconclusive and further studies are required to clarify the theme [11,14,16,21,22]. Moreover, it is interesting to select a fractionated method to compare the same total duration, the same intervals of rest but with different number of repetitions. Thus, the present research aimed to study the effects on internal and external loads resulting from the application of continuous and fractionated methods in SSGs in soccer training. It was hypothesized that the fractionated method, characterized by same total duration and same interval rest, but with different recovery times, induces a higher internal and external load and that the increase in the number of repetitions in the fractionated method raises the internal and external load compared to the continuous method.

## 2. Materials and Methods

### 2.1. Experimental Approach to the Problem

A cross-sectional field study was used to verify the differences between the continuous and fractionated methods with respect to internal and external load. Players were previously familiar with the different SSG formats and the material used. The study was conducted for four weeks with two days rest after the team’s official game and after a recovery session, to avoid the onset of fatigue. The study always took place on the same field and the 20 players participated in all data collection sessions. The players were distributed into two teams based on skill level and playing position to homogenize the competitive level. The teams did not change during the study. During each session and after a standard 15-min warm-up [23], one of the four SSG formats was applied, with several balls distributed throughout the field, ensuring that play continued quickly whenever the ball left the field [24]. To best control for circadian variations on the measured variables, all games were performed at the same time during the day (17:00–19:00) and, during these sessions, the average temperature recorded was 20 °C.

### 2.2. Subjects

Twenty male professional Portuguese soccer players (age: 25.2 ± 6.1 years; experience: 11.1 ± 4.2 years; height: 176.2 ± 7.3 cm; weight: 75.1 ± 6.7 kg) participated in the study during the 2018/2019 season. Their standard training involves four sessions per week (each lasting around 90 min), in addition to a competitive match. Participants were informed of the study design and its requirements, as well as the possible benefits and risks, and gave their consent prior to the start of the study in accordance with the principles of the Declaration of Helsinki for the study in humans. The study was approved by the local ethical committee (University of Beira Interior).

### 2.3. Small-sided Conditioned Games

All SSGs were composed of a 5 × 5 player format with a constant area of 40 m × 40 m. Four SSG formats were used in randomized order: one continuous T1 (1 × 24 min) and three fractionated methods, namely T2 (2 × 12 min), T3 (4 × 6 min) and T4 (6 × 4 min), with 2-min recovery between repetitions. No specific verbal instructions were provided before, during, or after the SSGs. Ten balls were placed around the pitch to ensure a quick repositioning if the ball in play went out of bounds. The SSGs followed official football rules with exception of offside. The aim of each game was to outscore the opponents.

### 2.4. Internal Load

Internal load was measured by recording HR (heart rate) with a GARMIN TM HR band (Garmin Ltd., Olathe, KS, USA) with a chest strap sensor [25]. The mean (Av.HR) and maximum (Max.HR) values recorded in each SSG format were considered for analysis.

### 2.5. External Load

External load was recorded using inertial WIMU TM devices (Real Track Systems, Almeria, Spain). The WIMU TM is composed of different sensors for motion analysis and tracking location under external conditions [26], demonstrating a high degree of accuracy [27]. Data were analyzed using the SPRO TM analysis program (RealTrack Systems, Almeria, Spain) and the displacement velocity was defined in four intervals of intensity: Very Low (0–1 m/s), Low (1–4 m/s), Moderate (4–5.5 m/s), and High/Very High (≥5.5 m/s).

### 2.6. Statistical Analysis

A descriptive analysis of the data was performed using standard deviations. Comparison between playing conditions was assessed by means of standardized mean differences calculated with combined variance and respective confidence intervals of 90% [28,29]. All assumptions were confirmed before data analysis. The limits for the statistics were 0.2, trivial; 0.6, small; 1.2, moderate; 2.0, large; and >2.0, very large [28]. Differences in means (i.e., T1 vs. T2, T1 vs. T3, T1 vs. T4, T2 vs. T3, T2 vs. T4, and T3 vs. T4) for each repetition and comparison of the entire total duration of 24 min were expressed in perception units with 90% confidence limits (CL). The smallest differences found were estimated from standardized units multiplied by 0.2. The probabilities were used to make a qualitative probabilistic mechanistic inference about the real effect; that is, if the effect probabilities were substantially higher and lower were both > 5%, the effect was reported as uncertain. Otherwise, the effect was clear and reported as the magnitude of the observed value. The scale was as follows: 25–75%, possible; 75–95%, likely; 95–99%, very likely; and >99%, most likely [28].

## 3. Results

**Figure 1:**
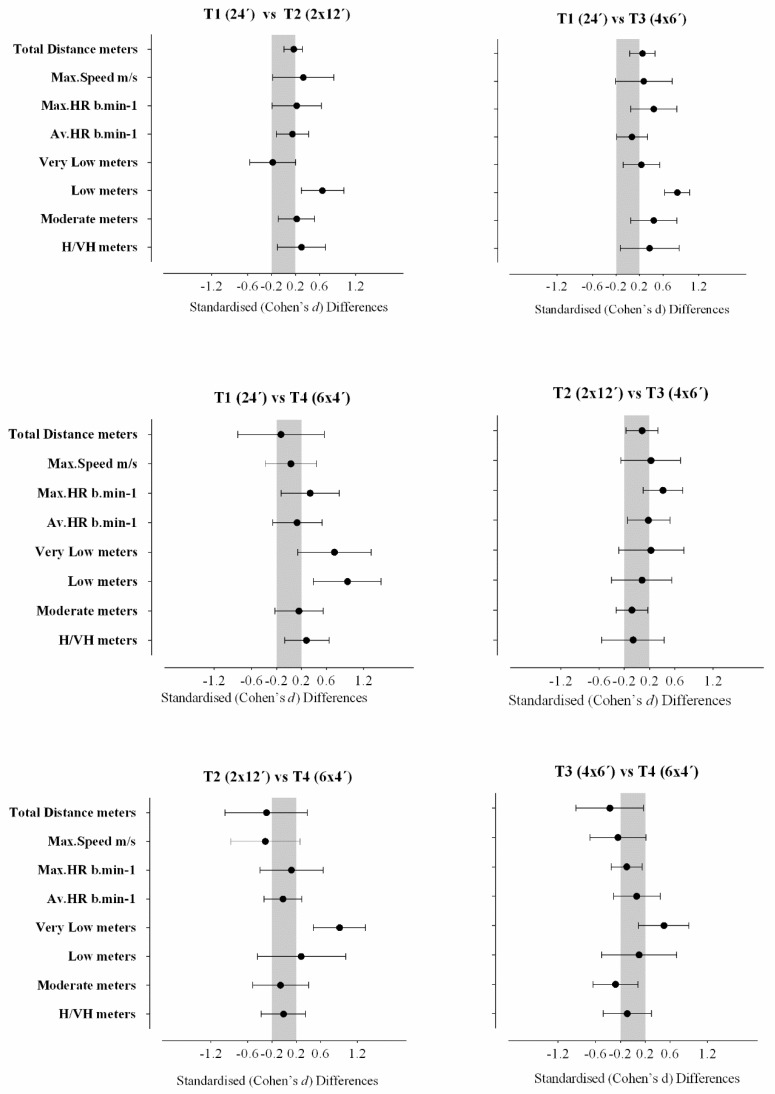
Standardized Cohen’s differences for comparative results of the T1 vs. T2, T1 vs. T3, T1 vs. T4, T2 vs. T3, T2 vs. T4, and T3 vs. T4 SSGs. Error bars indicate uncertainty in true mean changes with 90% confidence intervals.

Table 1 and Figure 1 show the variations in internal and external load between SSG formats T1 vs. T2, T1 vs. T3, T1 vs. T4, T2 vs. T3, T2 vs. T4, and T3 vs. T4. Overall, the fractionated method revealed a higher impact on the external load and subtle changes in the internal load of the players. It is apparent that, for the same time of exercise, the higher was the number of repetitions, the more internal load was imposed on the players.

### 3.1. Internal Load

The result of the internal load analyses revealed that the Max.HR of the players showed a possible increase of 4.7 ± 4.1 and 0.6 ± 5.0 (small effect) when comparing T1 vs. T3 and T1 vs. T4, respectively. However, a possible decrease of −1.1 ± 2.6 (trivial effect) was demonstrated by comparing T3 vs. T4. The Av. HR of the players revealed a possible 2.8 ± 5.0 increase (trivial effect) for T1 vs. T4.

### 3.2. External load

The results of total distance revealed possible increases of 79.1 ± 74.7 (trivial effect) and 116.8 ± 100.3 (small effect) for T1 vs. T2 and T1 vs. T3, respectively, and a possible decrease of −176.3 ± 257 (small effect) for T3 vs. T4. Regarding the maximum speed, a possible increase of 0.7 ± 1.0 (small effect) for T1 vs. T2 methods was observed.

Analysis of the very low-intensity travel speed revealed possible 15.0 ± 20.0 (small effect), 47.0 ± 38.2 (moderate effect), 26.7 ± 20.1 (small effect), and 32.0 ± 26.0 (small effect) increases and a most likely 58.7 ± 27.5 (moderate effect) increase for T1 vs. T3, T1 vs. T4, T2 vs. T3, T3 vs. T4 and T2 vs. T4, respectively.

Similarly, the low-intensity displacement velocity analysis revealed most likely increases of 168.7 ± 92.5 (moderate effect) and 241.7 ± 140.0 (moderate effect) and a possible increase of 46.7 ± 86, 2 (trivial effect) for T1 vs. T2, T1 vs. T4, and T2 vs. T3, respectively.

Analysis of moderate-intensity displacement velocity revealed a possible increase of 13.6 ± 19 (small effect) and a likely increase of 27.5 ± 24.6 (small effect) for T1 vs. T2 and T1 vs. T3, respectively. In addition, a possible reduction of 17.5 ± 22.3 (small effect) was revealed for T3 vs. T4.

Analysis of high-intensity displacement velocity revealed possible increases of 4.8 ± 6.5 (small effect), 6.1 ± 8.1 (small effect), and 4.6 ± 5.7 (small effect) for T1 vs. T2, T1 vs. T3, and T1 vs. T4, respectively.

## 4. Discussion

Overall, the use of the continuous method seems to present the tendency of less physical impact on the internal and external loads compared to the fractionated method. In addition, the increase in the number of exercise repetitions in the fractionated method was found to increase the external load compared to when using the continuous method. This latter method presented the tendency of the decreased in the distances travelled with different intensities. Regarding the HR responses, the data were trivially different, suggesting punctual variations between methods.

### 4.1. Internal Load

HR analysis revealed differences in Max.HR when comparing T1 vs. T3 and T1 vs. T4 formats, suggesting that it may be conditioned using the fractionated method, rather than the continuous method. Thus, evidence was provided in our study that the fractionated method performed by short repetitions (e.g., 4 min) induces further changes in Max.HR. Emphasizing the differences between continuous or fractionated methods, differences were found in a 3 × 3 format SSG study where longer repetitions (3 × 6 min/2 min rest) decreased Max.HR compared to shorter repetitions (3 × 2 min/2 min rest) [14]. Our results appear to reinforce the suggestion that the increase in total recovery time between exercises allow players to reach a higher intensity during exercises. This proposal was supported by previous studies when the results of Max.HR were crossed with intensity displacement velocity during the exercises, because the Max.HR were related to the increase in the pace of the game and the high-intensity actions of players [20].

Despite the considered variations in Max.HR responses, it is difficult to quantify the internal load variation based only on the use of continuous or fractionated methods, and other variables may be useful in future studies. However, the results appear to indicate that variations in Max.HR seems to be related to the use of specific 4 × 6 min and 6 × 4 min fractionated methods, thus increasing HR compared to the other longer fractionated (2 × 12 min) and continuous (1 × 24 min) methods used. These data suggest that fractionated methods can to induce a higher internal training load and rest periods between repetitions can be useful in recovery, allowing for increased physical responses in subsequent repetitions.

However, the analysis of Av.HR of the present study and some results of previous studies seem to present some divergent data. In a study conducted with national junior soccer players, no differences in physiological responses (internal and external training load) between continuous and fractioned methods were observed [30]. Similarly, Hill-Hass et al. [19] found no differences in physiological responses between the use of continuous and fractionated methods during SSG. These results are supported by a recent study, where three different sets of players performed exercises by both continuous and fractionated methods, and the physiological responses remained constant regardless of the training method used [9]. In addition, higher internal training load during continuous SSG performance are described, compared to the fractionated method [9]. Furthermore, the Av.HR results found in the present study are also somewhat contradictory. These differences are even more evident if we compare our findings with what was previously reported by Montgomery et al. [31] where high correlations between training load and HR responses were described. Our study does not follow this pattern because players’ physical responses appear to be higher in the fractionated method (higher Max.HR) but Av.HR responses tends to remain constant between the two training methods. Differences in protocol design and the fact that the HR may be sensitive to these differences may explain these results.

### 4.2. External Load

The results of external load related to the intensity of the displacements performed revealed that there is a tendency (from “possible” to “likely”) for higher values in the different displacement variables speeds by the use of the fractionated method compared to the continuous training method. The differences found may be due to the additional passive rest period between each repetition, which has a beneficial impact on delaying the impact of fatigue on players. This ability may have contributed to an improved physiological recovery of the body, including phosphocreatine resynthesis, the removal of metabolic by-products, and immobilized potassium in the muscle [1,32,33]. The results suggest that the rest period between 2 and 4 min was adequate for maintaining high intensity levels and maximizing energy phosphates as the primary energy source during exercise [34]. In addition, it has been previously shown that testosterone and cortisol respond to metabolic stress associated with SSGs, and some authors suggest that these hormonal changes may affect performance [35,36]. Thus, it is important to ensure an optimal total duration of exercise, with the number of repetitions and time of each one being correctly adjusted to avoid acute responses of the above hormones.

Regarding the total distance travelled, a higher total distance was identified in two formats performed in the fractionated method (T2 and T3, with a “possible effect”) compared to the continuous method. These data are in agreement with what was previously described by Hill-Haas et al. [19], who showed evidence of an increased total distance travelled during fractionated compared to continuous methods. These findings suggest that the continuous method tends to reduce the physical loads imposed on players, a result that can be explained based on the rest periods used in the fractionated method. However, when comparing the three fractionated methods, it can be inferred that, for this variable, the 4 × 6 min fractionated model presents a higher level of variation, specifically in the distance travelled. These data suggest that the exercise fractionation should not be too long or too short in relation to the total time, suggesting that 1/4 of the total exercise time per repetition is sufficient to guarantee high levels of physical demands consistently. This approach appears to contribute to the optimization of energy systems that support high-intensity explosive actions [37].

The maximum speeds between the methods do not seem to change significantly. The data indicate that the ability of players to reach high speeds is independent of the use of continuous or fractionated methods, possibly because the field size is reduced (40 m × 40 m), making it impossible for players to reach higher speeds. In the future, variations in the internal and external loads during SSGs performed by both continuous and fractionated methods in other game formats, with different manipulations of rules and constraints, may be analyzed to develop and clarify the theme.

This study highlights the importance of the coach’s choice when performing exercise by continuous or fractional methods. Coaches can manipulate this variable in order to manage the effect of exercise fatigue and increase or decrease exercise training load. For example, if the coach wants to maintain high physical performance and high training load responses in order to prepare players for a game’s demands, they should choose the fractional method of exercise with short repetitions. However, if the coach wishes to carefully manage the players’ efforts (e.g., post-competition muscle regeneration training) and decrease the response to the training load, they should use continuous exercise. If the coach wants to create an exercise with a lower training load, allowing players to focus more on learning other components over the duration, it would be more appropriate to select a continuous exercise (for example, 24 min). However, if the goal is to constantly provide adaptations to the game environment, highlighting what occurs during the game, the exercise should be performed in shorter repetitions (e.g.,4 × 6 min). Future studies should use the potential of this research to provide coaches with additional information, such as the impact on tactical behavior resulting from the application of both methods.

## 5. Conclusions

Application of SSGs by the fractionated method results in higher internal (small increments) and external (except very low intensities) loads. If trainers are seeking higher internal and external loads in a 5 × 5 SSG situation, the fractionated method would be the most appropriate one because continuous and longer exercise durations appear to be directly linked to a decrease in internal and external loads. However, it is important to note that the choice of one method always depends on the coach’s specific goals for the training session because there are numerous possibilities where both methods can be beneficial for performance enhancement. In addition, the increase in the number of exercise repetitions in the fractionated method seems to increase the external load compared to when using the continuous method during the same time of exercise duration. HR monitoring does not appear to be a suitable variable for assessing SSG load or intensity.

## Figures and Tables

**Table 1:** Descriptive statistics on the different condition variables.

Variables	T1 24 min Game	T2 2 × 12 min Game	T3 4 × 6 min Game	T4 6 × 4 min Game	Change in Mean (%; 90% CL)
Total distance meters	2254.5 ± 167.1	2333.60 ± 116.7	2371.28 ± 283.4	2194.9 ± 839.12	(a) 79.1; ±74.7 *
					(b) 116.8; ±100.3 *
					(c) −59.6; ±327.6
					(d) 37.7; ±118.3
					(e) −138.7; ±319.3
					(f) −176.3; ±257.0 *
Max. Speed m/s	6.05 ± 0.52	6.24 ± 0.59	6.20 ± 0.49	6.06 ± 0.54	(a) 0.7; ±1.0 *
					(b) 0.5; ±1.0
					(c) 0.1; ±0.8
					(d) −0.1; ±1.0
					(e) −0.6; ±1.1
					(f) −0.5; ±0.9
Max.HR b.min^−1^	181.95 ± 9.07	184.3 ± 10.03	186.60 ± 10.55	185.55 ± 11.16	(a) 2.4; ±4.4
					(b) 4.7; ±4.1 **
					(c) 3.6; ±5.0 *
					(d) 2.3; ±5.0
					(e) 1.3; ±5.5
					(f) −1.1; ±2.6 *
Av.HR b.min^−1^	152.7 ± 18.20	155.45 ± 16.90	154.00 ± 19.23	155.15 ± 15.16	(a) 2.8; ±5.0 *
					(b) 1.3; ±4.7
					(c) 2.5; ±7.3
					(d) −1.5; ±4.5
					(e) −0.3; ±5.6
					(f) 1.2; ±6.9
Very Low meters	262.06 ± 32.03	250.33 ± 50.95	277.06 ± 55.26	309.06 ± 93.41	(a) −11.7; ±24.4
					(b) 15.0; ±20.0 *
					(c) 47.0; ±38.2 **
					(d)26.7; ±20.1 **
					(e) 58.7; ±27.5 ***
					(f) 32.0; ±26.0 **
Low meters	1822.10 ± 176.05	1990.80 ± 171.27	2037 ± 180.68	2063.83 ± 389.43	(a) 168.7; ±92.5 ***
					(b) 215;.4 ±55.7
					(c) 241.7; ±140.0 ***
					(d) 46.7; ±86.2 *
					(e) 73.0; ±187.3
					(f) 26.3; ±153.9
Moderate meters	143.95 ± 45.66	157.52 ± 58.25	171.43 ± 65.74	153.88 ± 68.25	(a) 13.6; ±19 *
					(b) 27.5; ±24.6 **
					(c) 9.9; ±24.6
					(d) 13.9; ±32.1
					(e) −3.6; ±28.9
					(f) −17.5; ±22.3 *
H/VH meters	12.19 ± 12.27	17.03 ± 15.94	18.26 ± 18.85	16.80 ± 14.42	(a) 4.8; ±6.5 *
					(b) 6.1; ±8.1 *
					(c) 4.6; ±5.7 *
					(d) 1.2; ±7.6
					(e) −0.2; ±5.9
					(f) −1.5; ±6.4

Note: Differences in means ((%); ±90% CL) are identified as: (a) T1 vs. T2; (b) T1 vs. T3; (c) T1 vs. T4; (d) T2 vs. T3; (e) T2 vs. T4; and (f) T3 vs. T4. Asterisks indicate the uncertainty in the true differences as follows: * possible, ** likely, and *** very likely.
